# Deep Learning-Based Identification of Dental Implant Systems from Two-Dimensional Radiographs

**DOI:** 10.3390/diagnostics16172877

**Published:** 2026-09-07

**Authors:** Alparslan Esen, Mustafa Üstün

**Affiliations:** Department of Oral and Maxillofacial Surgery, Faculty of Dentistry, University of Necmettin Erbakan, 42090 Konya, Turkey; alparslanesen@gmail.com

**Keywords:** implant brand identification, convolutional neural network, panoramic radiography, transfer learning, computer-aided diagnosis

## Abstract

**Background/Objectives:** Dental implants are a reliable treatment for tooth loss, but identifying the implant brand when patient records are unavailable remains a clinical challenge that complicates prosthetic repair and complication management. This study aimed to develop and evaluate a deep learning-based system for automated identification of dental implant brands from panoramic and periapical radiographs. **Methods:** In this retrospective study, anonymized radiographs containing implants of twelve known brands were obtained from the archives of Necmettin Erbakan University Faculty of Dentistry. A two-stage pipeline was employed: a YOLOv11 detector first localized and cropped the implant regions, after which an EfficientNetV2-M convolutional neural network, fine-tuned via transfer learning, classified the implant brand. Class imbalance was addressed through offline and online data augmentation. **Results:** On the held-out test set of 531 implant crops spanning twelve brands, the classifier achieved an overall accuracy of 96.23% (95% CI 94.5–97.7%), a macro-averaged F1-score of 0.953, and a macro-averaged ROC-AUC of 0.991; the complete pipeline evaluated end to end on detector-predicted crops reached 96.0% match-conditional implant-level accuracy, corresponding to a precision-aware end-to-end identification F1-score of 88.2% (precision 81.6%, recall 96.0%) when all predicted boxes, including false detections, were counted. Grad-CAM analysis, including misclassified and low-confidence cases, indicated that predictions were based on clinically meaningful implant morphology. **Conclusions:** These findings indicate that the proposed two-stage approach provides accurate and interpretable implant brand identification, supporting its potential as a clinical decision support tool.

## 1. Introduction

Dental implants have become a highly predictable procedure in treating edentulous cases, with an estimated survival rate of more than 90% over ten years and beyond [1]. The efficiency of dental implants depends heavily on their unique design properties, including threads, necks, and surfaces. These features vary greatly between different implant manufacturers and directly influence osseointegration [2]. Due to persistent need, the market has developed into a complex domain, including over 2000 implant systems with minimal differences in morphology [3,4]. Knowing the exact brand of an implant is highly important when dealing with problems like screw loosening or fracture of the fixture because special components should be used [5]; however, sometimes, this information is unknown because patients do not remember their dental implants, or medical records are lost or scattered among clinics [6]. In these situations, it is necessary to resort to radiographic evaluation of the implant, which takes much time and is very difficult due to inter-observer variability, especially for rare and discontinued implant systems [7].

Recently developed techniques in the field of artificial intelligence (AI), especially deep learning (DL) based on convolutional neural networks (CNNs), have revolutionized image analysis by facilitating the automatic extraction of hierarchical and discriminative features without the need for any hand-crafted descriptors from raw images [8,9,10]. Landmark architectures such as ResNet and the Inception family have rapidly evolved in large-scale image classification [11,12]. Thanks to the incorporation of transfer learning, similar DL approaches have reached expert-level accuracy in a variety of diagnostic applications ranging from breast cancer histopathology to malaria and epidemic pathogen detection, skin cancer classification, and finally COVID-19 detection from radiographs [13,14,15]. Motivated by these successes, deep learning was employed to develop a fully automated system for brand identification of dental implants: Sukegawa et al. achieved 93.5% classification accuracy using a fine-tuned VGG16 network with 11 different brands [3], while later works have expanded the scope of the problem by using multi-brand datasets, various CNN backbone architectures, and object detection frameworks [6,7]. Despite this progress, two limitations recur throughout the literature: classification accuracy tends to decline as the number of target brands increases [16], and most models operate as opaque “black boxes” that provide little insight into the features underlying their predictions, thereby limiting clinical interpretability and trust. The need for transparent and accountable AI has been increasingly emphasized as a prerequisite for its safe and ethical deployment in clinical practice [17,18].

Accordingly, the goal of this study is to build a deep learning framework with two stages for automatic recognition of dental implant types through panoramic and periapical images.

## 2. Materials and Methods

In this study, a deep learning-based method was proposed for the automatic identification of dental implant brands, a task highly relevant in the field of oral and maxillofacial surgery. This research was conducted retrospectively utilizing panoramic and periapical radiographs collected from implant surgeries performed at Necmettin Erbakan University Faculty of Dentistry between January 2015 and March 2026. The proposed methodology involves a two-stage process: in the first stage, implant regions are detected within the panoramic and periapical radiographs using the YOLOv11m [19,20] object detection model; in the second stage, the detected implant regions are cropped from the original high-resolution radiographs and classified by a convolutional neural network (CNN) architecture pre-trained on the ImageNet dataset and fine-tuned via transfer learning. Details regarding data acquisition, preprocessing, model architectures, and training procedures are presented in the following subsections.

For this purpose, the Google Colab platform was used. Model training was performed on an NVIDIA H100 GPU (80 GB HBM3) using the PyTorch 2.10.0 framework with CUDA 12.8. A fixed random seed (42) was used for the data split, model weight initialization, and data augmentation to ensure reproducibility (Supplementary Table S2).

### 2.1. Data Acquisition and Preprocessing

The study was designed and reported in accordance with the applicable items of the STARD 2015 and CLAIM (Checklist for Artificial Intelligence in Medical Imaging) reporting guidelines for diagnostic accuracy and AI-in-imaging studies.

The panoramic and periapical images of the implants surgically applied during the specified period were accessed from the faculty’s records. All radiographs used in this study were captured using the Morita Veraviewepocs 3D R100P digital panoramic X ray device (J. Morita Corp., Kyoto, Japan) and saved in PNG format. Standard manufacturer-recommended imaging protocols (70 kVp, 8 mA, 7.4 s) were consistently applied during the radiographic procedures.

A total of 5130 implant annotations were obtained for the 1502 radiographs, which consisted of 322 periapical and 1180 panoramic radiographs. This set included implants from twelve different implant systems. In this context, location annotations of the implants were performed manually in the original panoramic and periapical radiographs using bounding boxes, and these annotation coordinates were used not only for training the detection algorithm but also for cropping the implant regions that were input in the classification process. To obtain optimal results for detection, the YOLOv11m model was trained by transfer learning and fine-tuning the hyperparameters. The implant dataset contained information on implant fixture, healing abutment, temporary prosthesis, and final prosthesis. The inclusion and exclusion criteria for the images were as follows:

Inclusion Criteria:

• Only radiographs taken with the Morita Veraviewepocs 3D R100P Digital Panoramic X ray Device at the Necmettin Erbakan University Faculty of Dentistry Department of Oral and Maxillofacial Radiology.
• Radiographs with sufficient diagnostic image quality, free of artifacts and superposition.
• Implant images that can be clearly perceived by the model after the cropping process.
• All implant images were included without selection among images with cover screws, healing abutments, or completed crowns.

Exclusion Criteria:

• Cropped images of implants with too low quality to be clearly perceived by the model.
• Radiographs with insufficient overall image quality or containing artifacts.

Image quality screening against these inclusion and exclusion criteria was performed by the annotating oral and maxillofacial specialist.

### 2.2. Ground Truth Annotation

The ground truth annotations were performed using the Roboflow platform. Every implant that could be seen on panoramic and periapical radiographs was annotated manually by a specialist in oral and maxillofacial surgery with a minimum of five years’ experience working in the field. For every implant, a bounding box was drawn around the whole implant structure visible on the radiograph, including its coronal, body, thread, and apical parts without the additional surrounding structures whenever possible. The bounding box coordinates were then saved in the YOLO format and used as the ground truth for training the model.

Each annotated implant was labeled with its brand from the verified clinical archive records, producing a ground truth dataset with two components: bounding box coordinates for localization and categorical labels for brand. These boxes served a dual purpose. They defined the target regions for YOLOv11m detection and, after expansion by a 15% margin to retain surrounding morphological context, provided implant-centered crops for EfficientNetV2-M classification. A single set of manual annotations thus supported both stages of the pipeline.

### 2.3. Proposed System

**Figure 1:**
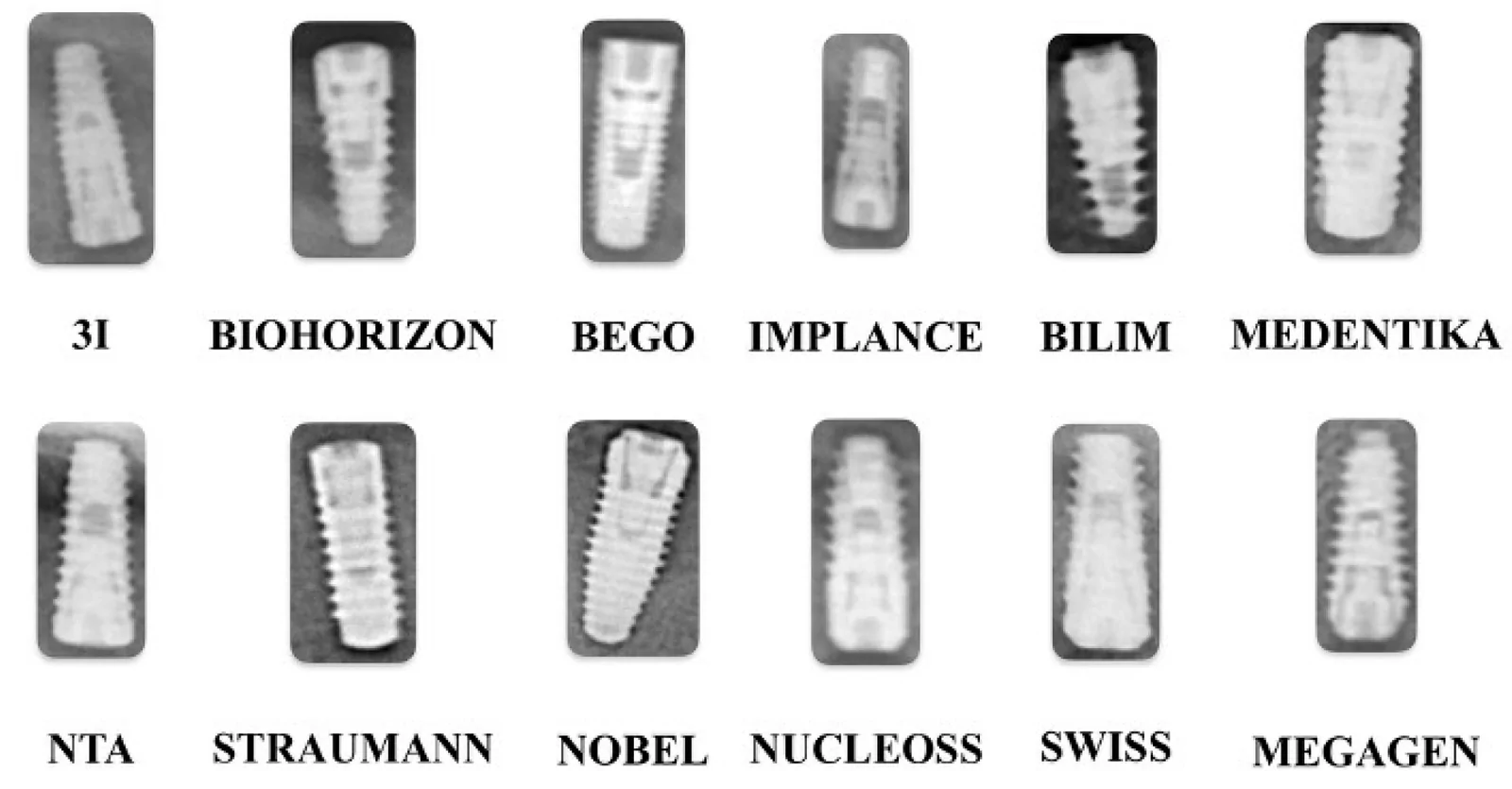
Representative implant examples, one per brand (12 brands), shown with their ground truth bounding box annotations used for training and evaluation. Boxes denote manually verified annotations rather than model predictions; detector confidence scores are therefore not displayed.

The proposed system focused on classifying 12 different implant brands (3I, BEGO, Bilimplant, BioHorizons, Implance, Medentika, MegaGen, Nobel Biocare, NTA, Nucleoss, Straumann, and Swiss). After curation, the final dataset comprised 1502 panoramic and periapical radiographs, on which a total of 5130 implants were annotated. The dataset was partitioned at the patient level: each radiograph was assigned a unique patient-based identifier as its file name, and these identifiers were used to ensure that all radiographs belonging to the same patient were allocated to the same subset, thereby preventing any same-patient overlap across the training, validation, and test sets. Within this patient-wise grouping, stratified sampling was applied using the dominant implant brand in each radiograph as the stratification criterion, and the radiographs were fully anonymized only after this partitioning step was completed. This yielded 1171 radiographs for training, 165 for validation, and 166 for testing—accounting for 4012 (78.2%), 587 (11.4%), and 531 (10.4%) implants, respectively. Prior to detection, all radiographs were resized to 640 × 640 pixels. To preserve class balance, we relied on online augmentation applied throughout YOLOv11m training; this involved geometric operations (rotation, translation, scaling, and flipping) and photometric adjustments (hue, saturation, and brightness), together with mosaic and mixup strategies. Examples of the implant classes examined in this study are shown in Figure 1.

**Figure 2:**
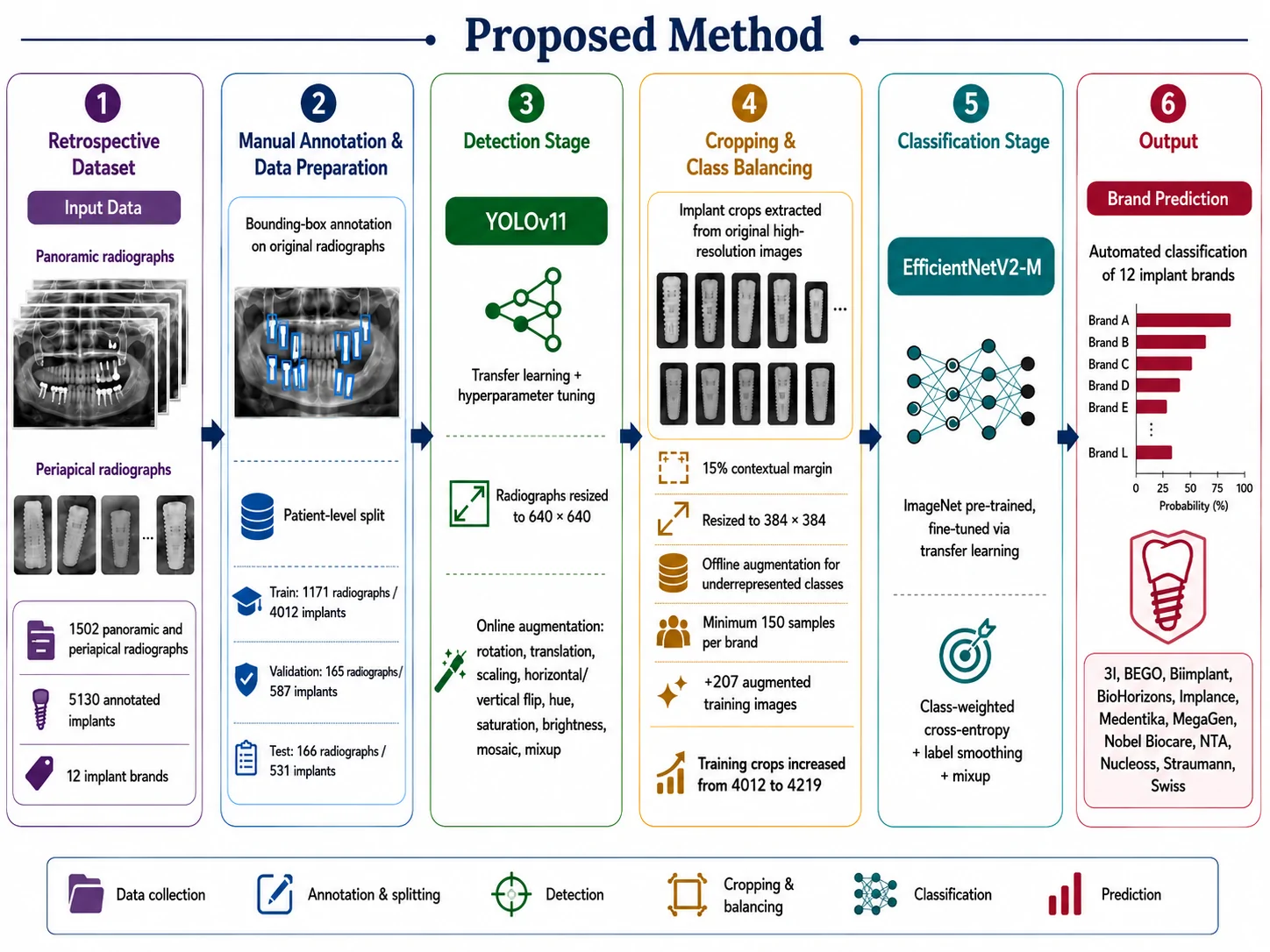
An overview of the proposed two-stage pipeline. Stage 1: YOLOv11m detects implant regions (confidence ≥ 0.25, NMS IoU 0.7); regions are cropped with a 15% contextual margin and resized to 384 × 384 px. Stage 2: EfficientNetV2-M assigns the implant brand. Class-weighted cross-entropy, label smoothing and mixup are applied during classifier training.

The proposed method is built on a two-stage architecture. In the first stage, YOLOv11m handles detection by locating implant regions within the panoramic and periapical radiographs. The crops produced at this step are then passed to an EfficientNetV2-M network—pre-trained on ImageNet and fine-tuned via transfer learning—which classifies the implant brands. This decoupled design allows brand classification to be performed on high-resolution implant crops; the rationale for separating localization from classification is examined in detail in Section 4.2. To counter the marked class imbalance, we incorporated several measures into the training process: a class-weighted cross-entropy loss, label smoothing, mixup regularization, and data augmentation. The overall workflow is illustrated in Figure 2.

Panoramic and periapical radiographs were pooled into a single dataset and modeled jointly: one detector and one classifier were trained across both modalities rather than training modality-specific models.

For the classification stage, each implant region was cropped from the original high-resolution radiograph using the ground truth coordinates with a 15% contextual margin and resized to 384 × 384 pixels, yielding implant crop sets identical in distribution to the patient-level split. Due to the unbalanced class distribution within the training set, offline data augmentation—including horizontal and vertical mirroring, 90-degree rotations, shift–scale–rotate transformations, brightness and contrast changes, and contrast-limited adaptive histogram equalization (CLAHE)—was applied to the under-represented classes in the training crops until each brand reached a minimum of 150 samples, generating 207 additional images and increasing the training set to 4219 implant crops (the complete set of augmentation transforms, parameters, and application probabilities is provided in Supplementary Table S1). No augmentation was applied to the validation or test images. Table 1 provides the brand-level distribution of the 5130 implants across the training, validation, and test subsets after training set augmentation.

An input resolution of 384 × 384 pixels was chosen for two reasons: it matches the native training resolution of EfficientNetV2-M (progressive training up to ~380 px), so the pre-trained filters operate at their optimized scale; and, after the 15% margin, the implant crops in our data have a median side of about 169 px (10th–90th percentile 112–254 px), so a 384 px input avoids down-sampling and preserves fine morphological detail (thread pitch, neck and apical form) at moderate computational cost. This 384 px input size was therefore an empirically and pragmatically motivated choice rather than a value proven optimal through a systematic input resolution ablation; such an ablation was not performed and is acknowledged as a remaining limitation.

### 2.4. Grad-CAM Analysis

Grad-CAM visualizations were generated from the gradients of the final convolutional layer of the EfficientNetV2-M network for representative correctly classified examples from each of the twelve implant brands. The resulting class-discriminative activation maps were used to qualitatively assess which image regions most influenced the model’s predictions; these findings are reported in Section 3.6.

### 2.5. Performance Evaluation

Model performance was evaluated using a confusion matrix generated by comparing predicted and actual classes over all 12 classes. The confusion matrix was the basis for calculating per-class accuracy, precision, recall, and F1 score, in addition to macro average and weighted average calculations, which were performed to account for class imbalance [21]. Furthermore, ROC-AUC and PR-AUC values were calculated for each of the 12 classes in a one-versus-rest setting, as these metrics provide different perspectives in evaluating the discrimination ability of a model and are especially useful in cases where there is class imbalance. Taken together, the confusion matrix-based metrics and the AUC analyses allowed for a thorough assessment of how well the EfficientNetV2-M model classified implants on the independent test set.

In addition to the classifier metrics, the YOLOv11m detector was evaluated separately on the test radiographs using precision, recall, mAP@0.5 and mAP@0.5:0.95, computed both class-agnostically (implant localization) and class-aware (brand). To reflect the behavior of the complete pipeline rather than performance conditional on a correct detection, we additionally report a precision-aware end-to-end operating point at the detector’s confidence threshold of 0.25. Every predicted box is counted: a correct brand identification requires both a valid localization (IoU ≥ 0.5 with a ground truth implant) and a correct brand label, whereas unmatched predicted boxes (false detections) are counted as identification errors. From these, identification precision, recall and F1 are computed over all predicted boxes. A complete end-to-end evaluation was also performed in which detector-predicted boxes (confidence ≥ 0.25, NMS IoU 0.7) were cropped from the original radiographs with the same 15% margin and classified; predictions were matched to ground truth at IoU ≥ 0.5 and unmatched ground truth implants were counted as errors. Per-class specificity and the Matthews correlation coefficient (MCC) were additionally computed. Uncertainty was quantified with 95% confidence intervals: overall metrics by bootstrap resampling of the test crops (2000 replicates) and per-class recall by Wilson score intervals.

## 3. Results

### 3.1. Dataset Characteristics and Final Image Distribution

The performance of the classifier was evaluated based on a separate test set including 531 samples of implants representing all 12 brands, following the patient-level split scheme presented in Section 2 (Table 1). The test set was kept class-imbalanced, retaining the imbalanced structure inherent to the original data, with the number of samples per brand varying from 4 (NTA) to 186 (BEGO). Some of the brands were evidently under-represented, for example SWISS (*n* = 11), BIOHORIZON (*n* = 18), and MEGAGEN (*n* = 26), among others. Since this imbalance is inherent to the distribution of the implant systems in clinical practice, it was specifically retained for the test phase to provide the evaluation under realistic conditions rather than artificial balance. This should be taken into account while considering the performance metrics for individual classes.

### 3.2. Model Training Performance

**Figure 3:**
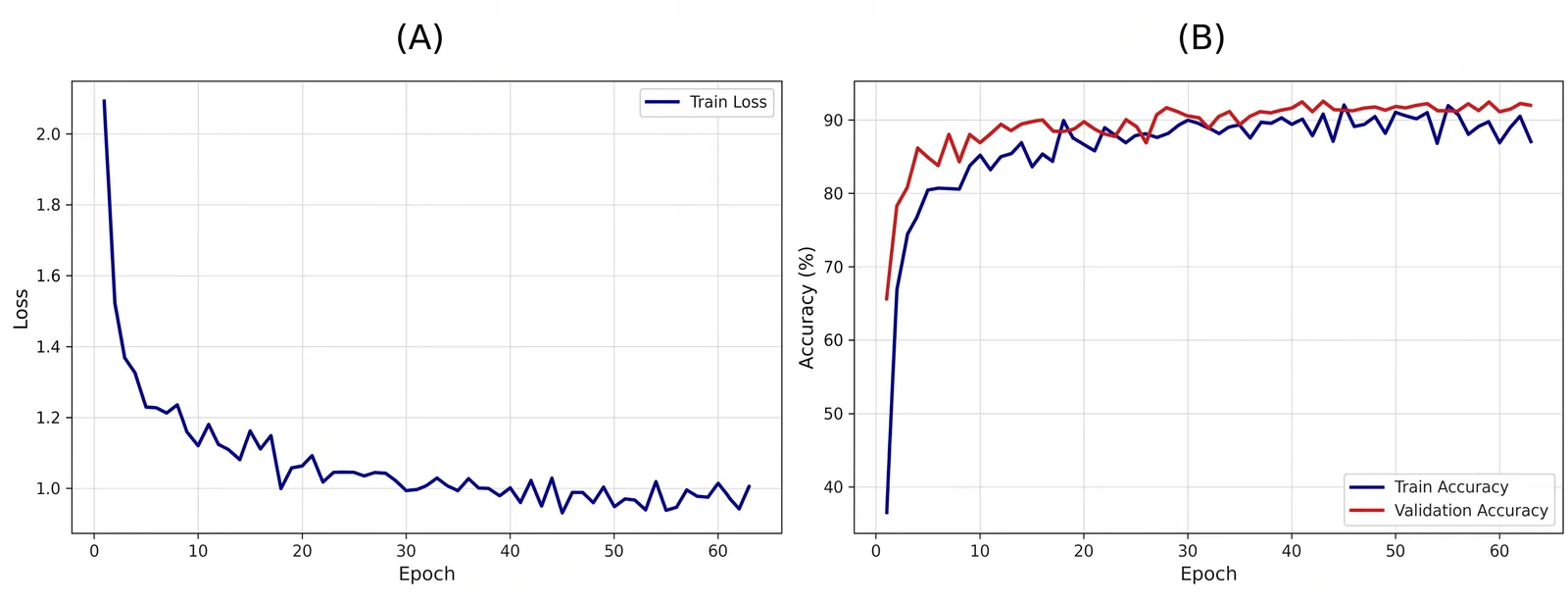
Classifier training dynamics. (**A**) Training loss across epochs. (**B**) Training and validation accuracy. The selected checkpoint (epoch 43, best validation accuracy) is marked; training continued to epoch 63 and was halted by early stopping (patience 20).

Figure 3 shows the learning curve of the EfficientNetV2-M model for 63 epochs of training. As shown in Figure 3A (loss curve), there was a sharp drop in the value of loss from about 2.10 to 1.20 in the first five epochs, which showed that the model adapted quickly to the classification problem at the start of fine-tuning. After epoch 20, the loss gradually dropped to a tight range of 0.93 to 1.00 and there were no further fluctuations in the curve.

The accuracy curves reinforced this picture (Figure 3B). Within the first ten epochs, training accuracy climbed from 36.1% to 80.6% and validation accuracy from 65.3% to 87.5%. After roughly epoch 30, both measures oscillated within a comparable 87% to 93% range, with validation accuracy generally sitting close to—or slightly above—training accuracy. This behavior is most likely attributable to the regularization strategies used during training, namely mixup and label smoothing, which make the training objective effectively harder than the unmodified validation set. The tight agreement between the two curves, with no widening generalization gap, indicates that significant overfitting did not occur and lends confidence to the subsequent test set evaluation.

### 3.3. Overall Classification Performance and Misclassification Patterns

**Figure 4:**
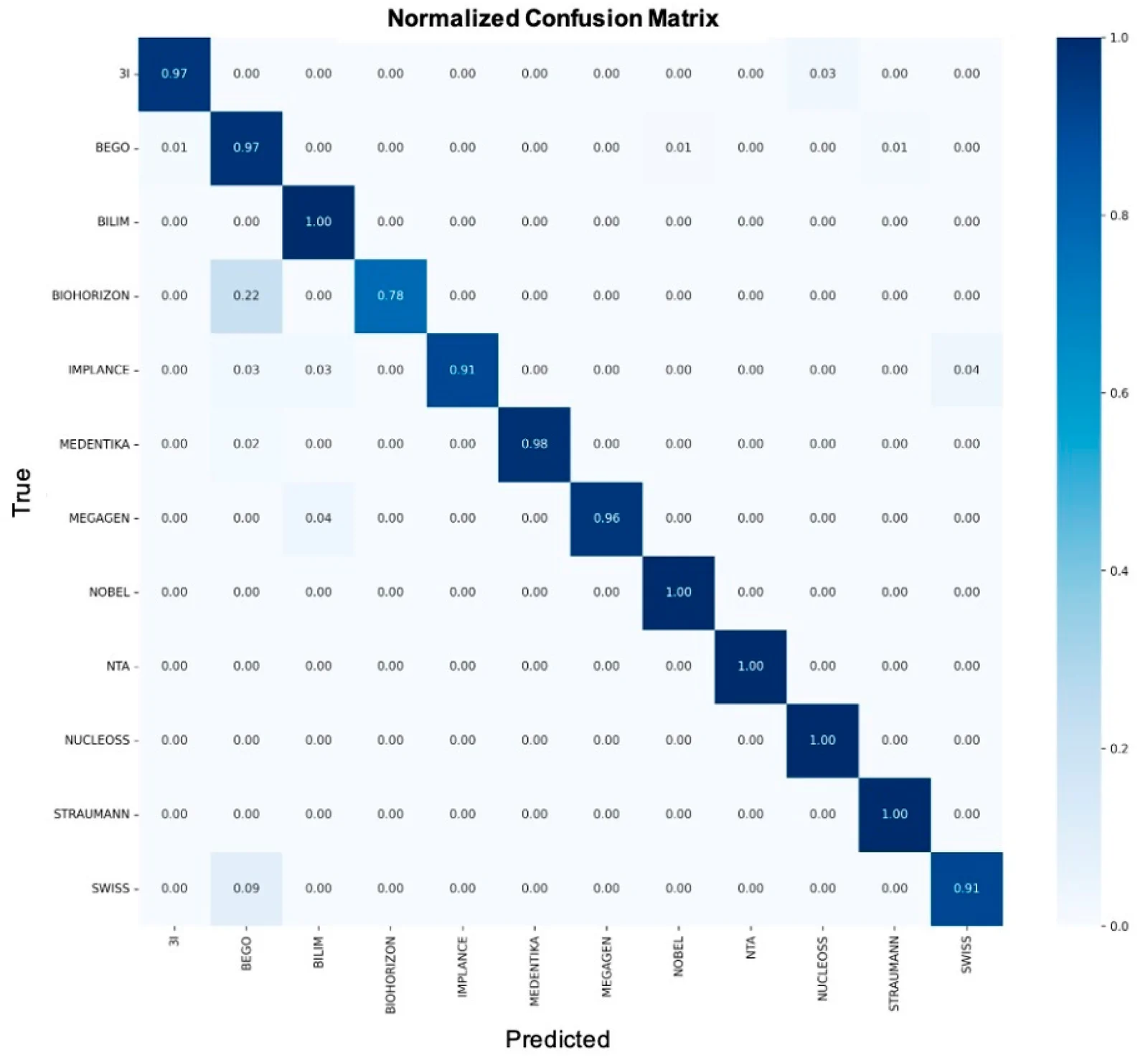
A confusion matrix of the EfficientNetV2-M classifier on the independent test set (*n* = 531). Each cell shows the raw count with the row-normalized proportion in parentheses; diagonal entries are correct classifications.

The overall performance of the proposed two-stage pipeline was evaluated on the held-out test set of 531 implant crops, on which the EfficientNetV2-M classifier achieved an overall accuracy of 96.23%. As shown in Figure 4, the confusion matrix was strongly diagonal-dominant across all 12 brands, indicating that the majority of samples were correctly classified despite the substantial variation in class size (from 4 instances for NTA to 186 for BEGO). Several brands—BILIM, NOBEL, NTA, NUCLEOSS, and STRAUMANN—had no misclassified test samples, although for the smallest of these (e.g., NTA, *n* = 4), the corresponding 95% confidence intervals are wide (Table 2); the remaining errors concentrated in a few specific class pairs rather than being broadly distributed across unrelated categories.

A total of 20 misclassifications were observed, corresponding to 3.77% of the test set. The most prominent involved BIOHORIZON, with 4 of 18 samples (22.2%) misclassified as BEGO—the largest single source of confusion. A secondary pattern affected IMPLANCE, accounting for seven errors distributed across SWISS (*n* = 3), BEGO (*n* = 2), and BILIM (*n* = 2). The remaining errors were isolated single misclassifications involving 3I, BEGO, MEDENTIKA, MEGAGEN, and SWISS. Overall, residual errors were mainly associated with implant systems of similar radiographic morphology—most notably BIOHORIZON and BEGO—rather than random confusion cross brands.

Across all classes, the macro-averaged specificity was 0.996 and the macro-averaged MCC was 0.950 (weighted 0.954), confirming that performance is maintained under the marked class imbalance (per-class values in Table 2). Test accuracy was equivalent across imaging modalities: 96.2% (461/479) on panoramic and 96.2% (50/52) on periapical radiographs; class-wise modality breakdowns are not reported owing to the small periapical sample.

### 3.4. ROC and Precision–Recall Curve Analysis

**Figure 5:**
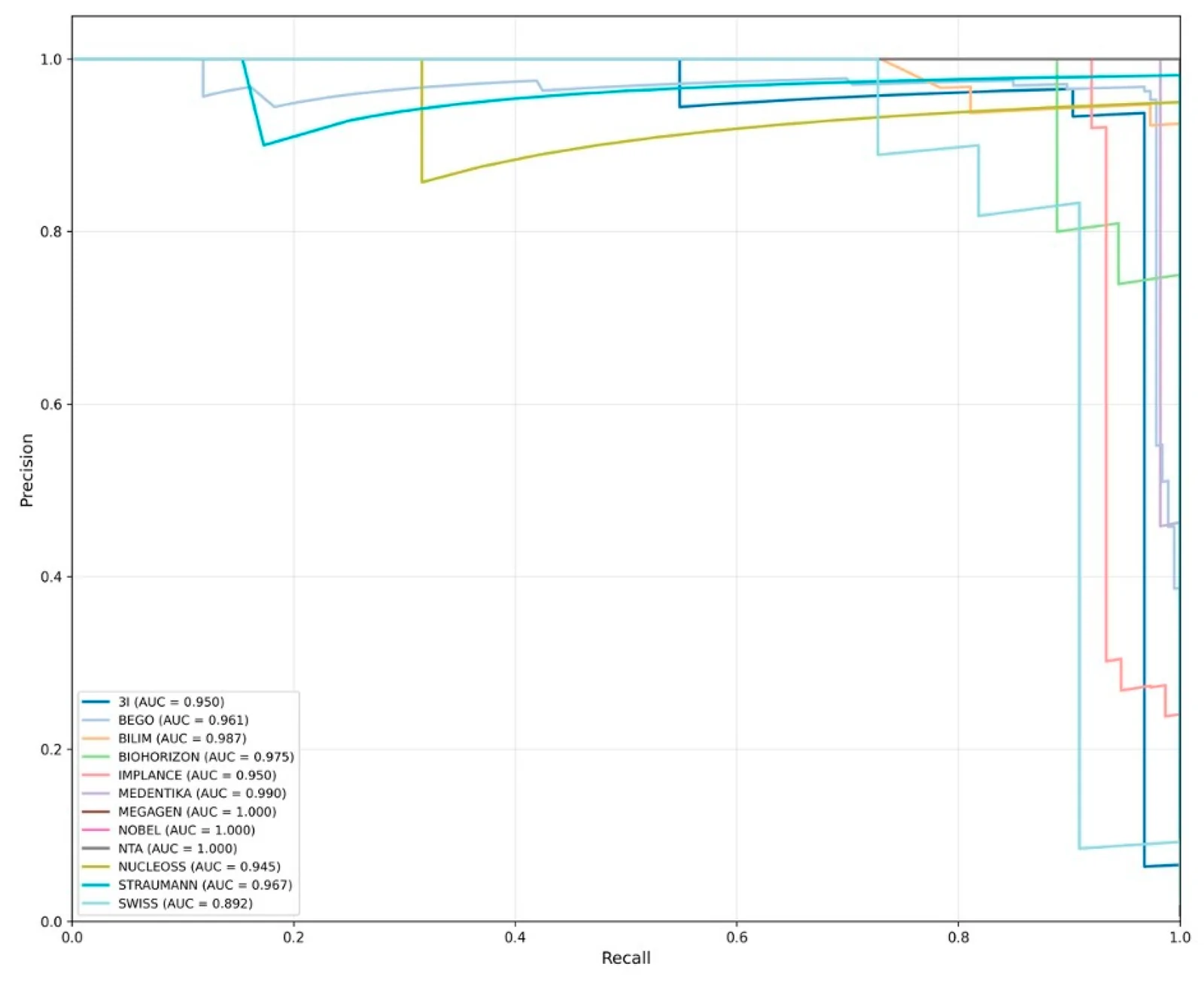
Representative Grad-CAM visualizations for correctly classified implants, one per brand. Activation maps were computed from the final convolutional block of EfficientNetV2-M and overlaid using the jet colormap (verified for grayscale/print readability). Warmer colors indicate regions of higher contribution to the predicted brand; activations concentrate on brand-discriminative morphology (thread pattern, neck and apical geometry).

The discriminative capacity of the classifier was evaluated using receiver operating characteristic (ROC) and precision–recall analyses for each brand in a one-versus-rest configuration. Class-wise ROC-AUC and PR-AUC values are presented in Table 2, and precision–recall curves for all classes are shown in Figure 5. The macro-averaged ROC-AUC was 0.991, indicating excellent overall separability across the 12 classes. Discrimination was nearly perfect for most brands, with BILIM, BIOHORIZON, MEDENTIKA, MEGAGEN, NOBEL, NTA, NUCLEOSS, and STRAUMANN achieving ROC-AUC values of 0.998 or higher. The values for 3I and IMPLANCE were slightly lower, at 0.971 each, but still indicate strong separability at most decision thresholds.

Precision–recall area under the curve (PR-AUC) is another metric of classification performance that is especially relevant when one class of samples is significantly under-represented compared to the other. The PR-AUC values varied from 0.892 in the case of SWISS implants to 1.000 for MEGAGEN, NOBEL, and NTA brands. Despite the smallest PR-AUC value, the ROC-AUC indicator of SWISS was quite large (0.981), which indicates the sensitivity of precision–recall properties of the classification algorithm to threshold changes and low presence in the test sample. NUCLEOSS and IMPLANCE had smaller PR-AUC values than the rest of the implant brands (0.945 and 0.950, respectively). In general, the high ROC-AUC and PR-AUC values demonstrate good discriminative performance of the classification algorithm for most brands, while SWISS, NUCLEOSS, and IMPLANCE are shown to be the most sensitive to sample distribution and threshold changes.

### 3.5. Confidence Threshold Analysis

To characterize the confidence–coverage trade-off, we computed coverage (the fraction of test predictions retained) as a function of the decision threshold (Supplementary Figure S1, Table S3). Coverage was 98.3% at a threshold of 0.5 but fell to 63.5% at 0.6, 42.0% at 0.8 and 12.1% at 0.9, whereas accuracy among the retained predictions changed very little (96.2–97.0%). Because label smoothing bounds the maximum softmax probability at approximately 0.95, and the median confidence of incorrect predictions (0.73) is close to that of correct predictions (0.78), raising the threshold discards many correct predictions while rejecting few errors. The default argmax operating point is therefore recommended, and probability calibration (e.g., temperature scaling) is identified as future work.

### 3.6. Qualitative Interpretation via Grad-CAM

**Figure 6:**
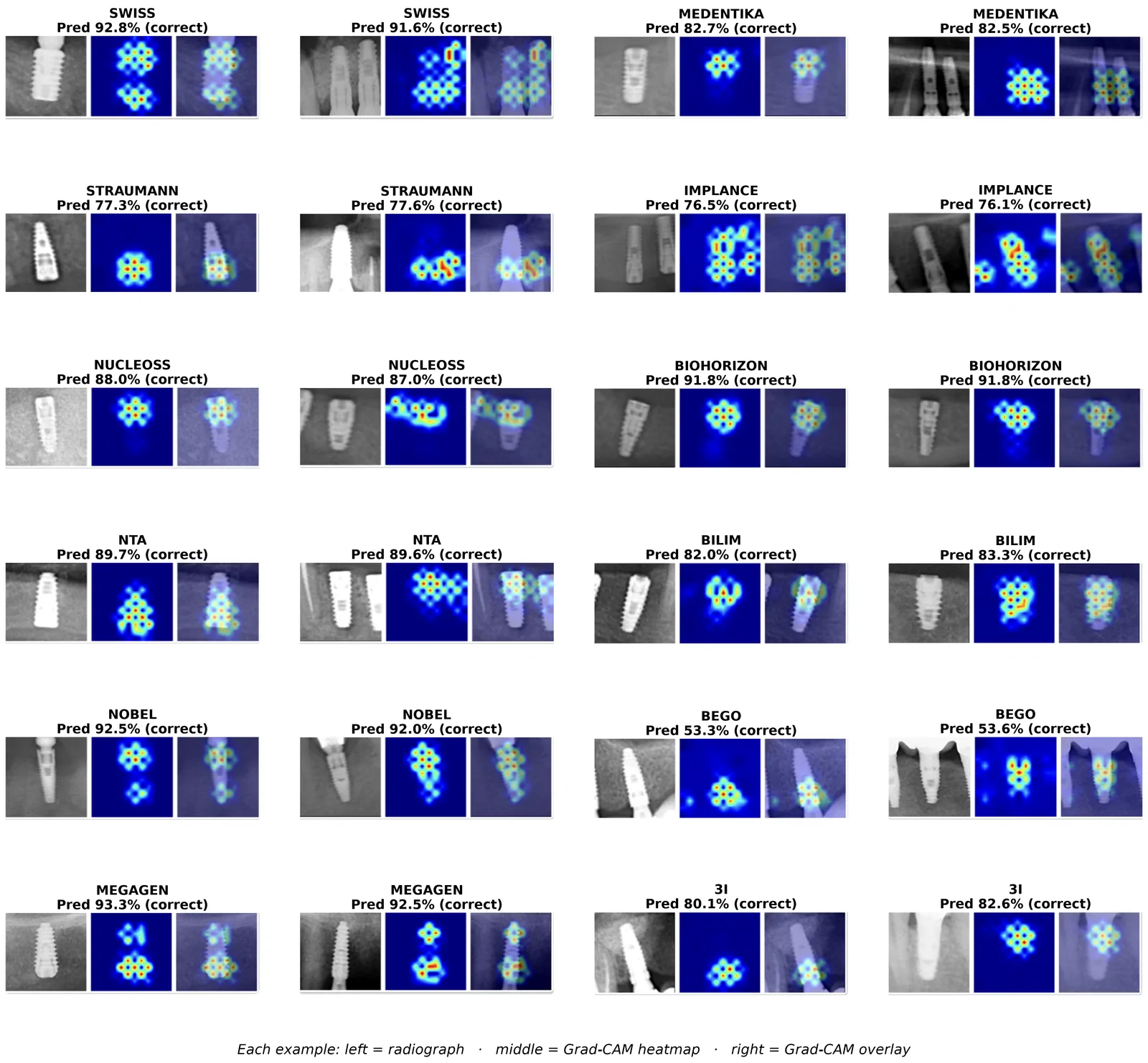
Representative Grad-CAM visualizations for correctly classified implants, one per brand. Activation maps were computed from the final convolutional block of EfficientNetV2-M and overlaid using the jet colormap (verified for grayscale/print readability). Warmer colors indicate regions contributing most to the predicted brand; activations concentrate on brand-discriminative morphology (thread pattern, neck and apical geometry).

In addition to the aforementioned quantitative measures of performance, Grad-CAM visualization was conducted on correctly classified representative examples for all 12 dental implant brands (Figure 6). Activation maps obtained from this procedure seemed to consistently focus on the morphologically relevant parts of the implant, such as coronal part, thread structure, body shape, and internal connection if visible. In most cases with only one implant in an image crop, activation was localized strictly to the target implant. Several crops containing multiple implants were found to have some activation in the surrounding areas or neighboring implants but the dominant feature of the results still pointed to the target implant.

Taken together, these qualitative observations indicate that the classifier arrived at its correct predictions chiefly by attending to radiographically meaningful design characteristics of the implant, rather than to unrelated background structures. Although confidence levels and activation patterns differed from one example to the next, the Grad-CAM outputs reinforced model interpretability by showing that attention was directed toward clinically plausible features relevant to distinguishing implant brands.

To avoid confirmation bias, Grad-CAM maps were additionally generated for all 20 misclassified test implants and the 12 lowest-confidence correct predictions (Supplementary Figure S2A,B). In the misclassified cases, the activations remained on the implant body and thread region rather than on background structures, indicating that errors arose from morphological similarity between brands (e.g., IMPLANCE↔SWISS, BIOHORIZON↔BEGO); in three cases, part of the activation fell on an adjacent implant within the crop, and in two BIOHORIZON cases the crop was of low resolution.

### 3.7. Detection Performance and End-to-End Evaluation

The YOLOv11m detector was evaluated on the held-out test radiographs (Table 3). For class-agnostic implant localization, it achieved a precision of 0.925, recall of 0.996, mAP@0.5 of 0.974 and mAP@0.5:0.95 of 0.746; the class-aware (12-brand) mAP@0.5 was 0.899 and mAP@0.5:0.95 was 0.713. Of the 531 test implants, 529 were localized (detection recall 99.6%; one IMPLANCE and one MEDENTIKA missed). The class-aware detector confusion matrix is provided in Supplementary Figure S3; its comparatively low diagonal values reflect the joint scoring of localization and brand assignment at the detection stage and should not be interpreted as the classifier’s accuracy.

In the true end-to-end evaluation, in which the classifier operated on detector-predicted crops rather than ground truth crops, classification accuracy was 96.4% on the predicted crops (versus 96.2% on ground truth crops), and the overall end-to-end implant-level accuracy was 96.0% (510/531). A single-stage YOLOv11m baseline, trained to detect and classify brands directly, reached 90.8% implant-level accuracy on the same split; the two-stage design improved recognition most for under-represented brands (e.g., NUCLEOSS 68.4%→100%, SWISS 72.7%→90.9%, NTA 75.0%→100%; Table 3). These results confirm that the reported classifier accuracy is not an artifact of using ground truth crops. When every predicted box is taken into account, the detector produced 625 boxes at a confidence threshold of 0.25 (529 matched, 96 unmatched/false), of which 510 corresponded to correct brand identification. This yields a precision-aware end-to-end identification precision of 81.6% (510/625), a recall of 96.0% (510/531) and an F1-score of 88.2%. The gap between the match-conditional accuracy (96.0%) and the precision-aware value reflects the false detections that would, in clinical use, constitute incorrect identifications; both figures are therefore reported so that the pipeline’s real-world behavior is not overstated.

### 3.8. Computational Efficiency

EfficientNetV2-M contains 52.9 M parameters (213 MB, FP32) and YOLOv11m 20.0 M parameters (40.5 MB). On an NVIDIA A100 GPU, detection took 17.6 ms per 640 × 640 radiograph and classification took 28.2 ms per crop at batch 1 (1.8 ms per crop at batch 32). A complete radiograph containing on average 3.8 detected implants was processed end to end in approximately 0.08 s with a peak GPU memory of 0.9 GB, indicating that routine clinical use does not require server-class hardware.

## 4. Discussion

### 4.1. Comparison with Prior Studies on Classification Performance

The automated identification of dental implant brands from radiographs has attracted considerable research attention in recent years, yet reported studies vary widely in dataset scale, the number of target classes, and methodological design, complicating direct comparison. A recent systematic review found that the accuracy of deep learning algorithms for implant detection and identification ranged from 67% to 98.5%—with most studies above 90%—and explicitly noted an inverse relationship between the number of implant classes and accuracy [16]. This trend is well illustrated by Kong et al. (2025), whose ensemble model classified 130 implant types at an accuracy of only 75.27%, while their subsequent object detection approach, reducing the task to 26 design categories, reached a mean average precision of up to 0.988 [7]. Such observations underscore that classification performance cannot be interpreted independently of task complexity.

Within more comparable ranges of class granularity, several studies have reported high performance on single-institution datasets. Sukegawa et al. (2020; 2021) reached 93.5% accuracy with a fine-tuned VGG16 network on 8859 implant images spanning 11 systems, and later showed that a multi-task framework could jointly classify implant brand and treatment stage across 12 brands without sacrificing accuracy [3,22]. More recently, the same group reported 91.46% accuracy by leveraging artificially generated training data to counter data scarcity [23], reflecting a wider shift toward augmentation-based strategies for under-represented classes. Using automated deep learning, Lee et al. (2020) [6] obtained an area under the curve of 0.954 for six implant systems, whereas Tiryaki et al. (2025) [24], whose dataset shares several brands with the present study, achieved a peak accuracy of 98.3% with a VGG-19 model, rising to 98.9% under a majority-voting fusion strategy, on 11,904 implant images of five brands. By contrast, larger multi-center datasets with greater class diversity have tended to report lower accuracies; Park et al. (2023) reported 88.53% accuracy across 27 implant types drawn from more than 150,000 images, attributing the reduction to inter-center acquisition variability and the larger number of classes [6,24,25].

Against this backdrop, the present study evaluated 5130 annotated implants representing 12 distinct brands and achieved a test accuracy of 96.23%, with a macro-averaged ROC-AUC of 0.991. This performance is particularly noteworthy given the well-documented decline in accuracy as the number of classes increases [16]; the proposed pipeline maintained accuracy above 96% while discriminating among twice as many brands as most high-performing single-institution studies [3,6,24] and remained substantially higher than the accuracy reported for the most class-diverse multi-center dataset [25]. Although models trained on five or six brands have reported marginally higher headline accuracies [23,24], such comparisons must account for the reduced classification difficulty inherent in smaller label spaces. The ability of the present two-stage approach to sustain high accuracy under greater class complexity suggests that the dedicated detection-then-classification design, combined with high-resolution implant crops, provides an effective strategy for fine-grained brand discrimination—a methodological aspect examined further in the following subsection.

A concise comparison of these prior studies with the present work—including dataset size, the number of brands/types, architecture and reported performance—is summarized in the prior-studies comparison illustrated Table 4.

### 4.2. Methodological Considerations of the Two-Stage Pipeline

An important characteristic of this work that distinguishes it from previous research in the field of implant identification lies in the separation of implant localization and implant brand recognition tasks into two independent stages instead of using a unified solution. As was mentioned previously, studies on this topic have either used image classification networks trained on manual implant region cropping [3,6], or single-stage object detection architectures such as YOLO and its modifications [4,7]. While single-stage object detectors enjoy benefits in terms of inference speed and end-to-end learning, their classification heads often operate on low-resolution feature maps, which may limit their capacity to recognize small differences between implants, like thread pitch, neck shape, and apical form. This study uses a different approach to implant classification: localization is performed with the help of YOLOv11m, and EfficientNetV2-M serves as a classifier of high-resolution crops.

This approach is in line with a growing number of studies that favor a cascaded approach for detecting and classifying implants. For example, in their work, Ariji et al. showed that a two-stage approach consisting of YOLOv7 for detection and EfficientNet for per-manufacturer implant type classification had achieved a recall score of 1.000 and an F1-score of 0.989 for detection, with metrics for classification above 0.92 for most types of implants [26]. This is consistent with our own end-to-end comparison (Section 3.7, Table 3), in which the two-stage design was associated with higher recognition accuracy than a single-stage baseline, particularly for under-represented brands. Furthermore, the choice of YOLO models for localization tasks is substantiated by numerous studies on the fast development and reliable performance of the YOLO family for medical object detection problems [27,28]. Moreover, the development of lightweight versions for real-time detection in resource-limited environments [29] also provides additional arguments in favor of the proposed architecture. The use of EfficientNetV2-M for classification relies on the proven capability of deep convolutional architectures to extract useful high-level features in radiographic imaging [3]. EfficientNetV2-M was specifically preferred for its favorable balance between accuracy and computational efficiency, achieved through fused-MBConv blocks and a compound scaling strategy that yields high parameter efficiency and fast, stable training compared to earlier networks such as ResNet [30]. Although attention-based architectures such as Vision Transformers, Swin Transformers, and the modernized ConvNeXt family have reported state-of-the-art results on large-scale benchmarks, they are generally more data-intensive and require substantially larger training sets to reach their full potential [31,32,33]. Given the moderate size of the present dataset, a parameter-efficient convolutional backbone therefore represented a more suitable choice, providing strong feature extraction and reliable transfer learning without such extensive data demands. Considering the excellent accuracy in Section 4.1, the presented reasons point to the advantage of a two-stage approach in providing increased performance for various implant brands and a modular structure of the model where each component can be easily updated.

### 4.3. Interpretability and Explainability of the Classification Model

Interpretability is yet another key requirement beyond accuracy for the successful adoption of deep learning algorithms in the clinic, as “black box” predictions offer little assistance in the clinical decision process and may hinder the confidence of clinicians in such systems. In order to solve this problem, the current study used a method named Grad-CAM that utilizes the gradients flowing into the last convolutional layer of the network to produce coarse localization maps for predicting the regions in the images that have the strongest effect on the particular prediction [34]. The produced activation maps always localized to morphological discriminators—the coronal part, threads shape, and apical morphology—rather than other anatomic structures or the background region. This suggests that the decisions made by the classifier were based on meaningful design attributes of the implant, as depicted in the images.

Explanations are still not a common component in the dental implant classifier evaluation literature, as most publications provide only performance measures without much analysis of the reasons behind their predictions [6,24,25]. However, this problem is becoming increasingly recognized as a drawback in the current research since transparency of artificial intelligence is considered crucial in ensuring ethical implementation of artificial intelligence techniques in dentistry [16]. The present study provides an example of how the implementation of Grad-CAM could help in increasing the transparency of explaining the decision-making process. It becomes especially important since interpretability may contribute to quality control of the prediction outcomes, helping the clinician understand why some images require additional analysis. By making the model’s reasoning visually accessible, Grad-CAM can strengthen clinicians’ trust in the system’s outputs and advocate its intended role as a clinical decision support tool rather than an opaque automated classifier. Moreover, activation maps that diverge toward the background or adjacent structures can serve as a practical signal that a given case warrants closer manual review, which is particularly valuable in a clinical setting where accountable decision-making is essential. These considerations align with recent frameworks calling for risk-averse, ethically governed medical AI [17,18].

### 4.4. Limitations and Clinical Applicability

A number of limitations should be addressed while analyzing the findings. First, there was substantial class imbalance within the dataset, whereby some of the brands had very few test cases, namely NTA (*n* = 4) and SWISS (*n* = 11). Class-wise metrics derived from such imbalanced datasets are statistically unreliable and make the findings generalizable only in the case of these systems to a certain extent. While the class-wise augmentation ensured an adequate number of training samples within each class, a low number of test samples meant that one misclassified image would lead to statistically significant changes within the respective per-class metrics; accordingly, per-class estimates for NTA (*n* = 4), SWISS (*n* = 11) and BIOHORIZON (*n* = 18) are imprecise, as reflected in their wide 95% confidence intervals (e.g., NTA recall 95% CI 0.51–1.00, Table 2), and should be interpreted as indicative rather than definitive. Such limitations have been acknowledged within previous studies that deal with dental implant identification, as brand popularity directly correlates with market presence [24,25].

A further limitation is that all 5130 bounding boxes were annotated by a single oral and maxillofacial specialist; formal inter-observer agreement analysis on a double-annotated subset was not performed and is left to future work. The high detection recall (99.6%) and the near-identical accuracy on detector-predicted versus ground truth crops provide indirect evidence of internally consistent annotation. We nonetheless emphasize that high detection recall is not a substitute for inter-observer agreement, and single-reader annotation remains an explicit limitation of this study; independent double annotation with formal reliability analysis is required in future work.

Another limitation is associated with using two-dimensional panoramic and periapical radiographs as the input images in the analysis, as these images might be prone to geometrical distortion, different levels of magnification, and the superimposition effect. These effects may blur out the morphological characteristics that are essential for distinguishing between implant brands [16]. In this regard, any remaining confusion between morphologically similar brands, such as BIOHORIZON and BEGO, might be due to the inherent limitations of two-dimensional imaging instead of model inadequacies. In fact, the literature on this topic acknowledges the incomplete nature of two-dimensional representations of osseous microarchitectures [35,36]. In addition, although panoramic and periapical radiographs were pooled, test accuracy was equivalent across modalities (96.2% for both; Section 3.3); a finer modality-stratified per-class analysis is limited by the small periapical sample and is left to future work.

Although periapical radiographs offer higher intrinsic spatial resolution than panoramic images, this did not translate into a measurable classification advantage, indicating that the brand-discriminative morphological features are already resolved at panoramic resolution for this task. Because the periapical test subset was small (*n* = 52), this equivalence should be read as the absence of a large modality effect rather than as evidence of exact parity, and a finer, adequately powered modality-stratified analysis remains a task for future work.

Regarding data partitioning, the dataset was split at the patient level (Section 2), so that no patient contributed radiographs to more than one subset; this design eliminates any same-patient information leakage between the training, validation, and test sets. It should nonetheless be noted that multiple implant crops originating from a single radiograph are not fully statistically independent; the reported bootstrap confidence intervals were therefore computed by resampling at the implant level to reflect this clustering.

These limitations suggest several areas where further research may prove efficient and provide clarity about the extent to which the pipeline should be used clinically. The three-dimensional modalities, specifically focusing on cone-beam computed tomography, overcome many limitations associated with two-dimensional radiographic images and have shown strong results when applied to deep learning-based implant segmentation and evaluation [36,37]. The integration of such modalities to the pipeline described above presents a viable way to gain more precise results.

A central limitation of this work is that both model development and evaluation relied on data from a single institution, acquired with a single imaging device under a fixed acquisition protocol. This single-center design constrains the external validity of the reported performance in three respects. First, the twelve brands examined here correspond to the systems routinely placed at our institution, so the observed brand distribution reflects local clinical practice rather than the global implant market; brands that are frequent elsewhere are absent from the dataset and therefore cannot be recognized by the current model. Second, because all radiographs originate from the same device and exposure settings (kVp, mA and exposure time), the model may have implicitly learned device-specific image characteristics—such as contrast, sharpness and noise texture—that would not transfer directly to radiographs obtained on other systems. Third, an independent external dataset could not be incorporated within the revision period: no public multi-brand radiographic dataset with matching, verified brand labels is currently available, and retrospectively sourced external radiographs would differ in acquisition device, exposure settings and label provenance factors that cannot be controlled after the fact and that would confound a fair external assessment. Therefore, the present findings should be regarded as a single-center proof of concept, and the model would need to be re-calibrated and re-validated before use in any other setting. Although the present results are encouraging, prospective multi-center validation using radiographs acquired from different imaging devices and institutions will be necessary to establish the robustness and clinical generalizability of the proposed framework, especially prior to routine clinical implementation.

Overall, the developed pipeline should be considered a clinical decision support system rather than an automated diagnostic algorithm, and it provides rapid preliminary identification to be confirmed through clinical records and expert judgment, consistent with current suggestions that artificial intelligence in implant dentistry should complement rather than replace human expertise [16].

In clinical use, the system is intended as a decision support module that receives a routine panoramic or periapical radiograph and returns, for each detected implant, a ranked brand suggestion with a confidence value for the clinician to confirm rather than a stand-alone diagnosis. Enumerated failure modes include morphologically similar brands, very small or low-resolution crops, and under-represented brands with wide confidence intervals; brands absent from the training set cannot be recognized, so the model must be re-trained as the local implant inventory changes. A controlled benchmark of transformer- and convolution-based backbones (Vision Transformer, Swin Transformer, ConvNeXt, DenseNet) under identical augmentation, class-weighting and resolution was beyond the scope of this revision and is planned as future work; EfficientNetV2-M was retained here for its favorable accuracy-to-cost balance on a dataset of this size.

## 5. Conclusions

In this study, a two-stage deep learning pipeline—YOLOv11m for implant localization and EfficientNetV2-M for brand classification—was proposed and evaluated on panoramic and periapical radiographs covering twelve implant brands. Despite the pronounced class imbalance, the approach achieved 96.2% classification accuracy given implant localization and 96.0% accuracy when evaluated end to end on detector-predicted crops, and Grad-CAM analysis indicated that its predictions were associated with clinically meaningful implant morphology rather than background features, supporting both the robustness and the explainability of the model.

Separating localization from classification, together with the use of high-resolution implant crops, proved to be an effective strategy for brand identification. These findings should, however, be regarded as a proof of concept: because the model was developed and tested on data from a single center and a single imaging device, external validation on independent, multi-center datasets acquired with different devices remains a necessary step before clinical use. Future work should also extend the framework to three-dimensional modalities such as cone-beam computed tomography and enrich the dataset with a wider, more balanced range of implant systems, including less frequent brands. Within these limits, the system is best positioned as a decision support tool that offers clinicians a rapid, interpretable preliminary identification—one to be confirmed against patient records and expert judgment—rather than a replacement for clinical evaluation.

## Figures and Tables

**Table 1:** The distribution of implants across the training, validation, and test partitions, showing the original and augmented training counts.

Class	Train (Original)	Train (Augmented)	Added	Validation	Test	Total
3I	327	327	0	42	31	400
BEGO	1370	1370	0	219	186	1775
BILIM	298	298	0	48	37	383
BIOHORIZON	120	150	+30	11	18	149
IMPLANCE	540	540	0	66	75	681
MEDENTIKA	300	300	0	52	56	408
MEGAGEN	121	150	+29	14	26	161
NOBEL	140	150	+10	15	16	171
NTA	49	150	+101	9	4	62
NUCLEOSS	208	208	0	30	19	257
STRAUMANN	426	426	0	73	52	551
SWISS	113	150	+37	8	11	132
TOTAL	4012	4219	+207	587	531	5130

Offline augmentation was applied only to under-represented training classes to reach a minimum of 150 crops per class (BIOHORIZON, MEGAGEN, NOBEL, NTA, and SWISS), adding 207 synthetic crops and increasing the training set from 4012 to 4219 crops. The validation and test sets were never augmented. The full annotated dataset comprised 5130 implants across 1502 radiographs.

**Table 2:** Per-class classification performance of the EfficientNetV2-M classifier on the held-out test set (531 implants), evaluated on ground truth crops. Recall and F1 are reported with 95% confidence intervals; specificity, Matthews correlation coefficient (MCC), and areas under the ROC and precision–recall curves are also given.

Class	Support	Precision	Recall	Recall 95% CI	Specificity	F1	F1 95% CI	MCC	ROC-AUC	PR-AUC
3I	31	0.938	0.968	0.838–0.994	0.996	0.952	0.883–1.000	0.950	0.971	0.950
BEGO	186	0.958	0.973	0.939–0.988	0.977	0.965	0.945–0.983	0.946	0.979	0.961
BILIM	37	0.925	1.000	0.906–1.000	0.994	0.961	0.911–1.000	0.959	0.999	0.987
BIOHORIZON	18	1.000	0.778	0.548–0.910	1.000	0.875	0.714–0.977	0.878	0.999	0.975
IMPLANCE	75	1.000	0.907	0.820–0.954	1.000	0.951	0.909–0.983	0.945	0.971	0.950
MEDENTIKA	56	1.000	0.982	0.906–0.997	1.000	0.991	0.970–1.000	0.990	0.998	0.990
MEGAGEN	26	1.000	0.962	0.811–0.993	1.000	0.980	0.933–1.000	0.980	1.000	1.000
NOBEL	16	0.941	1.000	0.806–1.000	0.998	0.970	0.889–1.000	0.969	1.000	1.000
NTA	4	1.000	1.000	0.510–1.000	1.000	1.000	1.000–1.000	1.000	1.000	1.000
NUCLEOSS	19	0.950	1.000	0.832–1.000	0.998	0.974	0.909–1.000	0.974	0.999	0.945
STRAUMANN	52	0.963	1.000	0.931–1.000	0.996	0.981	0.949–1.000	0.979	0.998	0.967
SWISS	11	0.769	0.909	0.623–0.984	0.994	0.833	0.615–0.966	0.833	0.981	0.892
**Macro avg**	**531**	**0.954**	**0.957**	**—**	**0.996**	**0.953**	**—**	**0.950**	**0.991**	**—**
**Weighted avg**	**531**	**0.964**	**0.962**	**—**	**0.991**	**0.962**	**—**	**0.954**	**—**	**—**

Support, the number of implants per brand in the test set; MCC, Matthews correlation coefficient; ROC-AUC, area under the receiver operating characteristic curve; PR-AUC, area under the precision–recall curve. 95% confidence intervals were obtained by implant-level bootstrap resampling. Wide intervals for the under-represented brands (e.g., NTA, *n* = 4; SWISS, *n* = 11; BIOHORIZON, *n* = 18) indicate that their per-class estimates should be interpreted as indicative rather than definitive. Overall accuracy was 96.23%.

**Table 3:** Detection performance of the two-stage pipeline, with a single-stage baseline comparison, on the held-out test set (531 implants). (**A**) Detection performance (precision, recall, mean average precision). (**B**) Classification and end-to-end accuracy, compared with a single-stage YOLOv11m baseline. (**C**) Per-brand detection recall and two-stage vs. single-stage accuracy.

(**A**)						
		**Precision**	**Recall**	**mAP@0.5**	**mAP@0.5:0.95**	
**Overall**						
Class-agnostic (implant localization)		0.925	0.996	0.974	0.746	
Class-aware (12 brands)		0.865	0.830	0.899	0.713	
**Per brand (class-aware)**						
3I		0.905	0.935	0.933	0.736	
BEGO		0.918	0.906	0.946	0.668	
BILIM		0.801	0.730	0.896	0.734	
BIOHORIZON		1.000	0.796	0.995	0.736	
IMPLANCE		0.955	0.853	0.943	0.768	
MEDENTIKA		0.963	0.857	0.940	0.766	
MEGAGEN		0.931	0.923	0.980	0.810	
NOBEL		0.878	0.904	0.965	0.754	
NTA		0.890	0.750	0.768	0.662	
NUCLEOSS		0.742	0.684	0.795	0.652	
STRAUMANN		0.789	0.923	0.871	0.645	
SWISS		0.603	0.694	0.751	0.620	
(**B**)						
**Overall Metric**				**Value**		
Implants in test set (*n*)				531		
Detection recall (IoU ≥ 0.5)				99.6% (529/531)		
Missed detections				2 (IMPLANCE 1/75, MEDENTIKA 1/56)		
Classifier accuracy on ground truth crops				96.23%		
Classifier accuracy on detected crops				96.41%		
End-to-end accuracy, two-stage (YOLOv11m→EfficientNetV2-M)				96.05%		
Single-stage YOLOv11m classification accuracy (baseline)				91.12%		
Single-stage YOLOv11m end-to-end accuracy (baseline)				90.77%		
**Precision-Aware End-to-End (All Predicted Boxes** **, conf 0.25)**						
Predicted boxes (matched + false)				625 (529 + 96)		
Identification precision				81.6% (510/625)		
Identification recall				96.0% (510/531)		
Identification F1				88.2%		
(**C**)						
**Class**	***n***	**Detected**	**Detection Recall**	**Two-Stage acc.**	**Single-Stage acc.**	**End-to-End acc. (Two-Stage)**
3I	31	31	100.0%	96.8%	96.8%	96.8%
BEGO	186	186	100.0%	97.3%	92.5%	97.3%
BILIM	37	37	100.0%	100.0%	91.9%	100.0%
BIOHORIZON	18	18	100.0%	88.9%	94.4%	88.9%
IMPLANCE	75	74	98.7%	90.5%	87.8%	89.3%
MEDENTIKA	56	55	98.2%	96.4%	89.1%	94.6%
MEGAGEN	26	26	100.0%	96.2%	92.3%	96.2%
NOBEL	16	16	100.0%	100.0%	100.0%	100.0%
NTA	4	4	100.0%	100.0%	75.0%	100.0%
NUCLEOSS	19	19	100.0%	100.0%	68.4%	100.0%
STRAUMANN	52	52	100.0%	100.0%	98.1%	100.0%
SWISS	11	11	100.0%	90.9%	72.7%	90.9%

mAP, mean average precision; acc., accuracy. Class-agnostic detection scores for implant localization only; class-aware detection requires both correct localization and correct brand assignment, hence its lower values. Two-stage accuracy invovles EfficientNetV2-M classification on the crops produced by the detector; end-to-end accuracy additionally reflects the two undetected implants. The single-stage baseline uses YOLOv11m alone for simultaneous detection and brand classification.

**Table 4:** Comparison with prior implant identification studies.

Study	Dataset Size	Brands/Types	Architecture	Performance
Lee et al. (2020) [6]	—	6 systems	Automated deep learning (CNN)	AUC 0.954
Sukegawa et al. (2020) [3]	8859 images	11 systems	Fine-tuned VGG16	93.5% acc
Sukegawa et al. (2021) [22]	—	12 brands	Multi-task CNN (brand + stage)	acc maintained vs. single-task
Sukegawa et al. (2025) [23]	+synthetic data	multiple	CNN + generative augmentation	91.46% acc
Park et al. (2023) [25]	>150,000 images	27 types	Deep CNN (multi-center)	88.53% acc
Tiryaki et al. (2025) [24]	11,904 images	5 brands	VGG-19 (+majority voting)	98.3% (98.9% fusion)
Kong et al. (2025) [7]	—	130 types/26 categories	Ensemble CNN/object detection	75.27% acc/mAP up to 0.988
Present study	1502 radiographs, 5130 implants	12 brands	Two-stage: YOLOv11m + EfficientNetV2-M	96.23% acc, macro ROC-AUC 0.991, end-to-end 96.0%

## Data Availability

The original contributions presented in this study are included in the article/Supplementary Materials. Further inquiries can be directed to the corresponding author.

## Supplementary Materials

The following supporting information can be downloaded at: https://www.mdpi.com/article/10.3390/diagnostics16172877/s1, Table S1. Data-augmentation parameters and probabilities; Table S2. Detector and classifier training configuration; Table S3. Coverage (retained prediction fraction) vs. confidence threshold; Figure S1. Confidence–coverage trade-off of the EfficientNetV2-M classifier on the test set. Coverage falls from 98.3% at a threshold of 0.5 to 12.1% at 0.9, while the accuracy of retained predictions stays within 96.2–97.0%. Tabulated values are given in Table S3; Figure S2A. Grad-CAM maps for all 20 misclassified test implants. Activations remained on the implant body and thread region rather than background, indicating that errors arose from morphological similarity between brands (e.g., IMPLANCE↔SWISS, BIOHORIZON↔BEGO); Figure S2B. Grad-CAM maps for the 12 lowest-confidence correct predictions. Even at low confidence, activations stayed localized to radiographically meaningful implant design features rather than background structures; Figure S3. Class-aware (12-brand) confusion matrix of the YOLOv11m detector on the test set. Its comparatively low diagonal values reflect the joint scoring of localization and brand assignment at the detection stage and should not be interpreted as the classifier’s accuracy.
