# Supplementary material for: Deep Learning-Based Identification of Dental Implant Systems from Two-Dimensional Radiographs
Source: Diagnostics (Basel). 2026 Sep 7;16(17):2877. doi: 10.3390/diagnostics16172877 (PMC13565568; doi:10.3390/diagnostics16172877)
Supplement: Supplementary file 1 [file diagnostics-16-02877-s001.zip › diagnostics-4491854-supplementary.pdf]

Supplementary Tables

Supplementary Table S1. Data-augmentation parameters and probabilities.

Stage	Transform	Parameters	p
**Offline (train crops of under-represented classes, to ≥150/class)**	HorizontalFlip	–	0.5
**Offline (train crops of under-represented classes, to ≥150/class)**	VerticalFlip	–	0.5
**Offline (train crops of under-represented classes, to ≥150/class)**	RandomRotate90	–	0.5
**Offline (train crops of under-represented classes, to ≥150/class)**	ShiftScaleRotate	shift 0.10, scale 0.15, rotate ±20°	0.7
**Offline (train crops of under-represented classes, to ≥150/class)**	RandomBrightnessContrast	brightness 0.2, contrast 0.2	0.5
**Offline (train crops of under-represented classes, to ≥150/class)**	CLAHE	clip limit 2.0	0.3
**Online (every training iteration, classifier)**	Resize 384×384	–	1.0
**Online (every training iteration, classifier)**	HorizontalFlip / VerticalFlip / RandomRotate90	–	0.5 each
**Online (every training iteration, classifier)**	ShiftScaleRotate	shift 0.10, scale 0.20, rotate ±25°	0.6
**Online (every training iteration, classifier)**	OneOf {RandomBrightnessContrast (0.2/0.2), CLAHE (clip 3.0)}	–	0.5
**Online (every training iteration, classifier)**	GaussianBlur	kernel 3–5	0.2
**Online (every training iteration, classifier)**	Mixup	α = 0.2	every batch
**Detector (YOLOv11m, built-in)**	HSV h/s/v	0.015 / 0.4 / 0.3	–
**Detector (YOLOv11m, built-in)**	rotation / translation / scale	±15° / 0.10 / 0.30	–
**Detector (YOLOv11m, built-in)**	fliplr / flipud	–	0.5 / 0.3
**Detector (YOLOv11m, built-in)**	mosaic / mixup	–	0.8 / 0.1 (mosaic disabled for last 10 epochs)

Supplementary Table S2. Detector and classifier training configuration.

Component	Parameter	Value
**Detector (YOLOv11m)**	Input resolution	640 × 640
**Detector (YOLOv11m)**	Initialization	COCO-pretrained
**Detector (YOLOv11m)**	Optimizer	AdamW (lr0 0.002, final-lr factor 0.01, weight decay 0.01, 5 warm-up epochs)
**Detector (YOLOv11m)**	Batch size	64
**Detector (YOLOv11m)**	Epochs / early stopping	max 150, patience 25 (stopped at 132; epoch-107 weights used)
**Detector (YOLOv11m)**	Loss weights	box 7.5 / cls 1.0 / dfl 1.5
**Detector (YOLOv11m)**	Inference conf / NMS-IoU	0.25 / 0.70
**Detector (YOLOv11m)**	Parameters	20.0 M
**Classifier (EfficientNetV2-M)**	Input resolution	384 × 384
**Classifier (EfficientNetV2-M)**	Initialization	ImageNet-pretrained (transfer learning)

**Classifier (EfficientNetV2-M)**	Optimizer	AdamW (lr 2×10^{-4}, weight decay 0.02)
**Classifier (EfficientNetV2-M)**	LR schedule	Cosine annealing (T = 80, η_min = 10^{-6})
**Classifier (EfficientNetV2-M)**	Batch size / precision	32 / mixed precision (AMP)
**Classifier (EfficientNetV2-M)**	Epochs / early stopping	max 80, patience 20 (best val at 43; stopped at 63; epoch-43 weights used)
**Classifier (EfficientNetV2-M)**	Loss	Class-weighted cross-entropy + label smoothing
**Classifier (EfficientNetV2-M)**	Regularization	Mixup (α = 0.2), inverse-frequency class weights
**Classifier (EfficientNetV2-M)**	Parameters	52.9 M
**Both**	Framework	PyTorch 2.10.0, CUDA 12.8
**Both**	Random seed	42 (fixed for data split, weight initialization, and augmentation)

Supplementary Table S3. Coverage (retained prediction fraction) vs confidence threshold.

Confidence threshold	Coverage (retained %)	Accuracy on retained	Weighted F1	Macro F1
**0.1**	100.0%	96.23%	0.962	0.953
**0.25**	99.8%	96.23%	0.962	0.953
**0.5**	98.3%	96.55%	0.966	0.958
**0.6**	63.5%	96.74%	0.962	0.889
**0.7**	63.1%	97.01%	0.965	0.891
**0.8**	42.0%	95.52%	0.936	0.808
**0.9**	12.1%	96.88%	0.954	0.653

Supplementary Figures

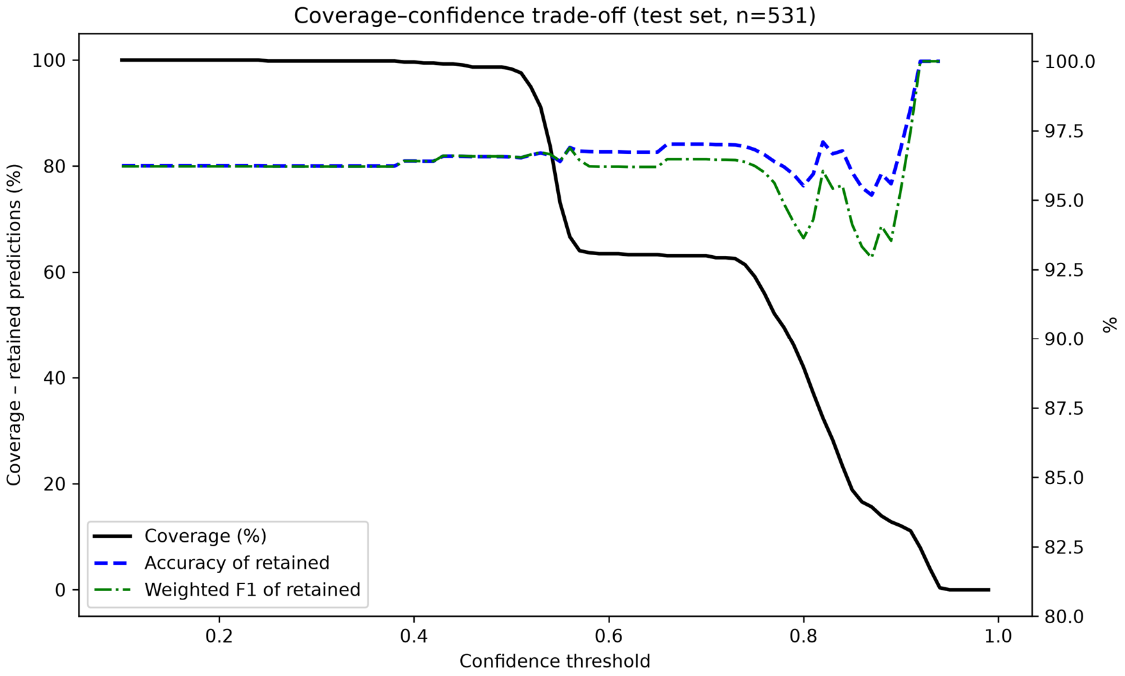

Supplemantary Figure S1: Confidence–coverage trade-off of the EfficientNetV2-M classifier on the test set. Coverage falls from 98.3% at a threshold of 0.5 to 12.1% at 0.9, while the accuracy of retained predictions stays within 96.2–97.0%. Tabulated values are given in Table S3

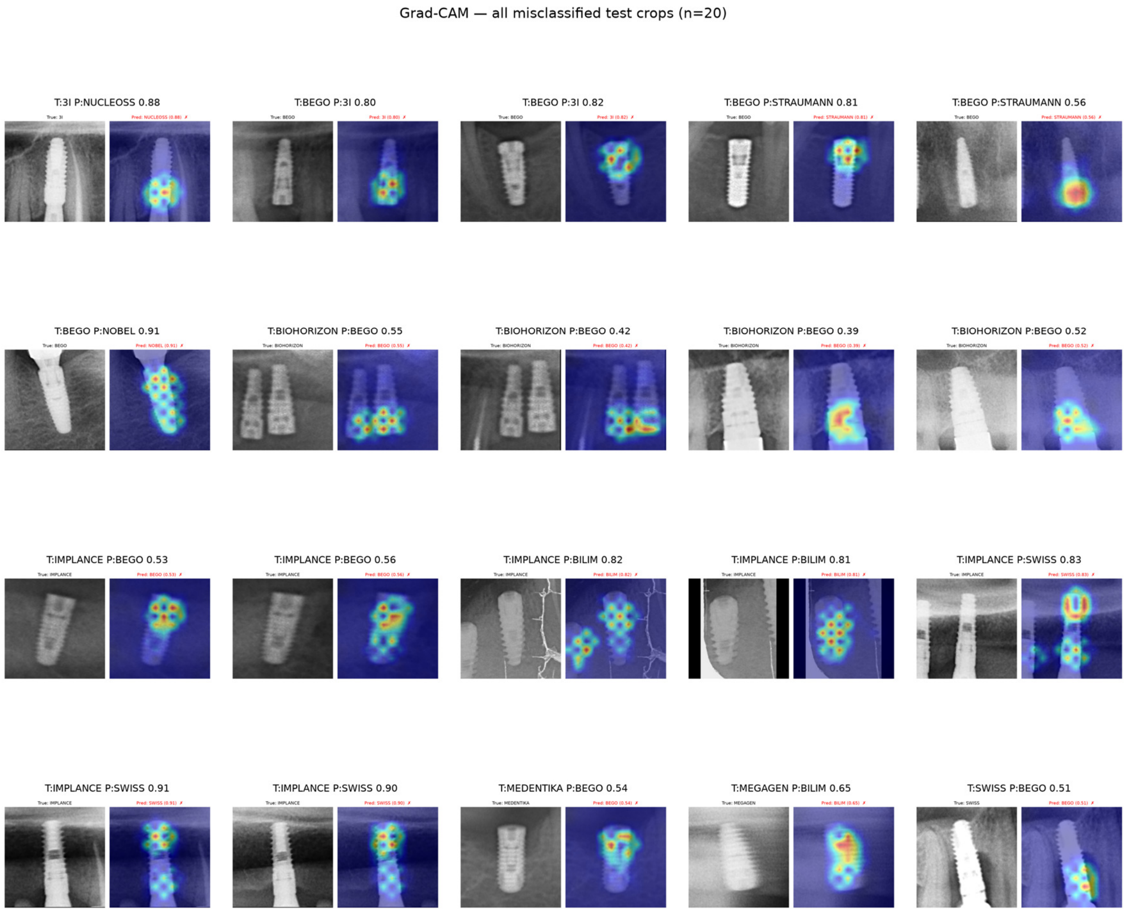

Supplemantary Figure S2A: Grad-CAM maps for all 20 misclassified test implants. Activations remained on the implant body and thread region rather than background, indicating that errors arose from morphological similarity between brands (e.g., IMPLANCE↔SWISS, BIOHORIZON↔BEGO)

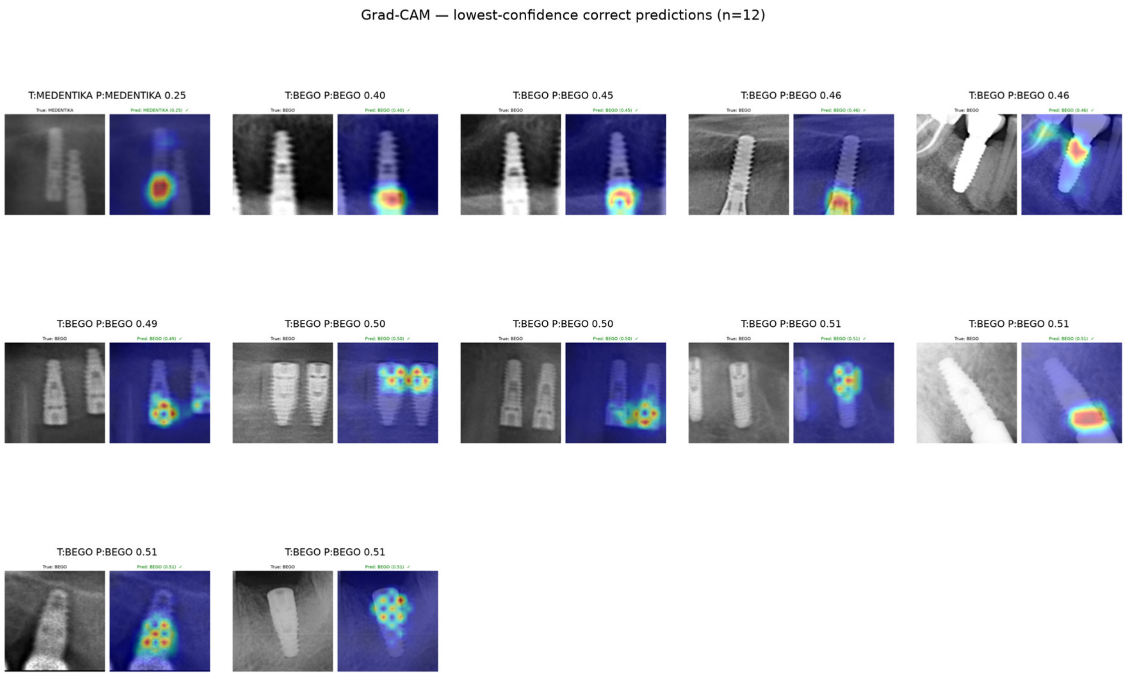

Supplemantary Figure S2B: Grad-CAM maps for the 12 lowest-confidence correct predictions. Even at low confidence, activations stayed localized to radiographically meaningful implant design features rather than background structures.

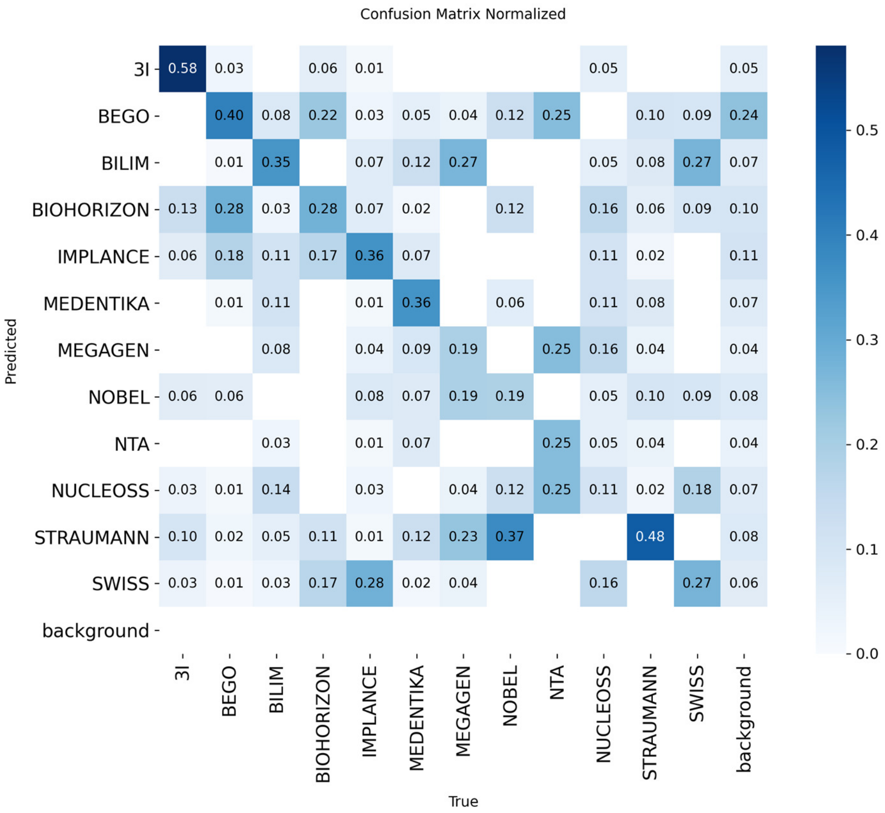

Supplemantary Figure S3: Class-aware (12-brand) confusion matrix of the YOLOv11m detector on the test set. Its comparatively low diagonal values reflect the joint scoring of localization and brand assignment at the detection stage and should not be interpreted as the classifier's accuracy
